# Male predominance in sports sudden cardiac arrest: a behavioural driver of risk?

**DOI:** 10.1093/europace/euag125

**Published:** 2026-06-26

**Authors:** Richard Chocron, Xavier Jouven

**Affiliations:** Université Paris Cité, Inserm, PARCC, Paris F-75015, France; Emergency Department, AP-HP, European Georges Pompidou Hospital, Paris F-75015, France; Université Paris Cité, Inserm, PARCC, Paris F-75015, France; Cardiology Department, AP-HP, European Georges Pompidou Hospital, Paris F-75015, France

## Abstract

**Graphical Abstract:**
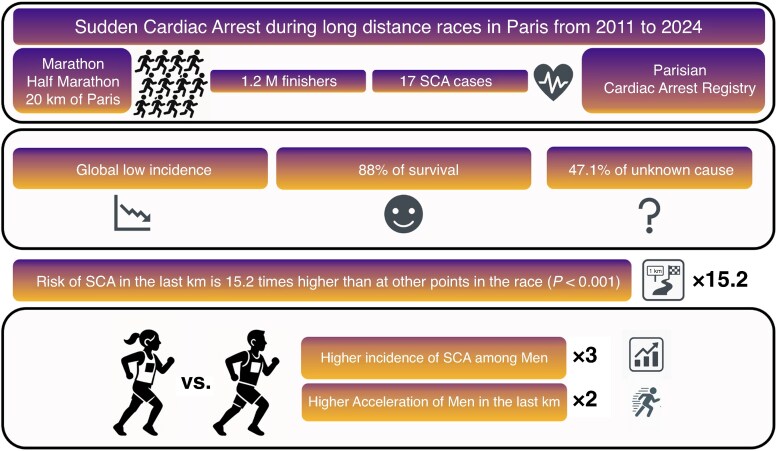

We thank Dr. Kato for his thoughtful comments on our article on sudden cardiac arrest (SCA) during endurance races.^1^

Our study was observational and not designed to establish causality. We could not assess pacing changes in runners who experienced SCA. Therefore, we cannot determine whether they accelerated more than finishers. However, several consistent patterns emerged. First, SCA cases showed a marked male predominance after adjustment for participation. Second, events were concentrated in the final kilometre. Third, among finishers, men were about twice as likely as women to accelerate in the last kilometre. Taken together, these findings suggest sex-related differences in responses to a period of maximal physiological stress, potentially reflecting different racing behaviours. These observations raise two key questions: why do men accelerate more in the final phase and does such final acceleration during a period of maximal physiological stress increase the risk of SCA?

The male predominance in sports-related SCA is consistently reported across endurance sports, team sports, and competitive athletics.^2^ Despite this consistency, no single physiological mechanism fully explains the magnitude of this disparity. Proposed factors, such as differences in myocardial electrophysiology, myocardial electrophysiology, autonomic regulation, hormonal environment, or arrhythmia susceptibility^3^ remain insufficient.^4^ Our study therefore explored a complementary and under-investigated hypothesis: behavioural response to effort and the tendency to push beyond prior exertion in the final phase of competition. We observed that male runners were more likely to increase pace in the last kilometre, suggesting a greater propensity to exceed physiological limits at peak stress. This behavioural patterns, described by Hsieh^5^ as ‘maladaptive perfectionism’, may contribute to heightened sympathetic activation and increased cardiac workload, potentially triggering malignant arrhythmias in susceptible individuals. Such mechanisms may extend beyond running to other sports involving a final maximal effort, though they remain difficult to quantify.

In conclusion, while our study does not establish causality, it highlights behavioural responses to extreme effort as a plausible contributing factor to the male predominance in sports-related SCA. These findings support further research into the interplay between physiological susceptibility, behavioural patterns, and sociocultural influences in exercise-related SCA.
